# Case Report: Pituitary Morphology and Function Are Preserved in Female Patients With Idiopathic Intracranial Hypertension Under Pharmacological Treatment

**DOI:** 10.3389/fendo.2020.613054

**Published:** 2021-01-08

**Authors:** Rossella Tozzi, Antonietta Moramarco, Mikiko Watanabe, Angela Balena, Alessandra Caputi, Elena Gangitano, Elisa Petrangeli, Stefania Mariani, Lucio Gnessi, Carla Lubrano

**Affiliations:** ^1^ Department of Molecular Medicine, Sapienza University of Rome, Rome, Italy; ^2^ Department of Sense Organs, Sapienza University of Rome, Rome, Italy; ^3^ Section of Medical Pathophysiology, Food Science and Endocrinology, Department of Experimental Medicine, Sapienza University of Rome, Rome, Italy

**Keywords:** IIH, *pseudotumor cerebri*, growth hormone, prolactin, empty sella, obesity, pituitary MRI, pituitary hormones

## Abstract

Idiopathic Intracranial Hypertension is a neurological disorder primarily affecting overweight women of childbearing age. It is often characterized by radiologic evidence of empty sella (ES), which is in turn frequently associated with pituitary dysfunction, with the somatotropic axis most commonly affected. No recent evidence is available relative to the presence of pituitary hormone deficiencies in adult patients with Idiopathic Intracranial Hypertension (IIH) under pharmacological therapy. We therefore explored pituitary function and morphology in a small cohort of female patients with IIH treated with acetazolamide. Fifteen female patients aged 42 ± 13 years with IIH lasting between 12 and 18 months were evaluated. All patients were affected by recurrent headaches in addition to visual changes of variable severity. IIH diagnosis was made after exclusion of other causes of raised intracranial pressure, and a specific ophthalmological evaluation was conducted to assess for the presence of papilledema. No particular endocrinological disturbances were detected during the enrolment visits, except for a high obesity prevalence (87%, BMI 35.16 ± 8.21 kg/m^2^), one case of total thyroidectomy for papillary thyroid carcinoma and two patients with irregular menses and mild hirsutism. All the participants underwent a pituitary MRI with contrast, and two different operators performed pituitary measurements in coronal and sagittal scans for morphologic assessment. Blood samples for the anterior pituitary axis evaluation were collected, and the somatotropic axis was further evaluated with a GHRH + Arginine test; other dynamic tests were performed in case of suspected hormonal deficiency. Despite ES being found in 73% of the patients, pituitary volume was preserved, ranging from 213.85 to 642.27mm^3^ (389.20 ± 125.53mm^3^); mean coronal pituitary height was 4.53 ± 1.33 mm. Overall, baseline anterior pituitary hormones levels were within normal ranges, and none of the patients with ES had an altered response to the GHRH + arginine stimulation test. We found one patient suffering from iatrogenic hyperthyroidism and two diagnosed with subclinical primary hypothyroidism due to Hashimoto’s thyroiditis. Two young patients were suspected of having polycystic ovary syndrome, and they were therefore further investigated. In conclusion, this case series shows that, despite the high prevalence of ES, the pituitary function of IIH patients treated with acetazolamide is preserved. To date, there is no evidence regarding the trend over time or upon treatment discontinuation in regard to the pituitary function of patients with IIH, and it is therefore not possible to infer whether our finding would be replicable in such settings. We therefore suggest an endocrine follow-up over time in order to monitor for potential pituitary dysfunction.

## Introduction

Idiopathic intracranial hypertension (IIH), also known as *pseudotumor cerebri*, is defined as an elevation of intracranial pressure with normal brain parenchyma appearance, absence of ventriculomegaly and no identifiable cause. Studies showed that the incidence of IIH is between 1 and 2 per 100,000 in adults, increasing to 3 per 100.000 in women and reaching 12–32 out of 100,000 in overweight women by childbearing age (1), suggesting that female hormones and pregnancy may represent a risk factor. Moreover, although obesity seems to be clearly associated with this condition, the pathophysiology underlying this link remains undetermined. Noteworthy, the only epidemiological study conducted in Italy showed a lower annual incidence rate (2.7/100,000) for overweight women in reproductive age (2). Patients with IIH show no alterations in neuroimaging studies other than total or partial empty sella (ES) being demonstrated in up to 76–85% of cases (3). This seems to be a consequence of the long-standing effects of increased intracranial pressure leading to a downward herniation of an arachnocele through the diaphragma sellae within the *sella turcica* (4). ES can be associated with impaired pituitary function, particularly in subjects with overweight or obesity (5): the reported prevalence is of 20–60%, and it often involves the GH/IGF-1 axis (6).

Only a few studies, mostly conducted in children, investigated the hormonal axes in patients affected by IIH, reporting isolated pituitary alterations such as GH deficiency (GHD) or central hypothyroidism (7–9). Full restoration of the pituitary function occurred upon treatment with acetazolamide or lumbo-peritoneal shunting (10, 11). No recent studies, instead, were conducted to evaluate the pituitary function in adult patients suffering from IIH (12, 13). Given the established correlation between stable or intermittent increase in intracranial pressure and ES and therefore a possible depression in pituitary function (5, 14), we aimed at studying the pituitary axis in relation to its morphology in a small cohort of young female patients affected by pseudotumor cerebri on pharmacological treatment.

## Case Series Description

Fifteen female patients, aged between 16 and 62 years, were consecutively recruited among those referring to the Department of Sense Organs, Sapienza University of Rome, between September 2017 and February 2019. All participants were asked to sign a written informed consent before enrolment (it was obtained from parents or legal tutors for patients under 18 years old). The study was conducted according to the principles of the 1964 Declaration of Helsinki and its later amendments. All patients suffered from recurrent headaches managed with symptomatic drugs occurring since about 6 months preceding the first evaluation. In addition, the patients reported visual changes of variable severity, with the most common complaint being transient central visual obscurations improving after the introduction of drug therapy, and no other neurological sign or symptom such as sixth cranial nerve palsies or pulsatile synchronous tinnitus.

**Figure 1:**
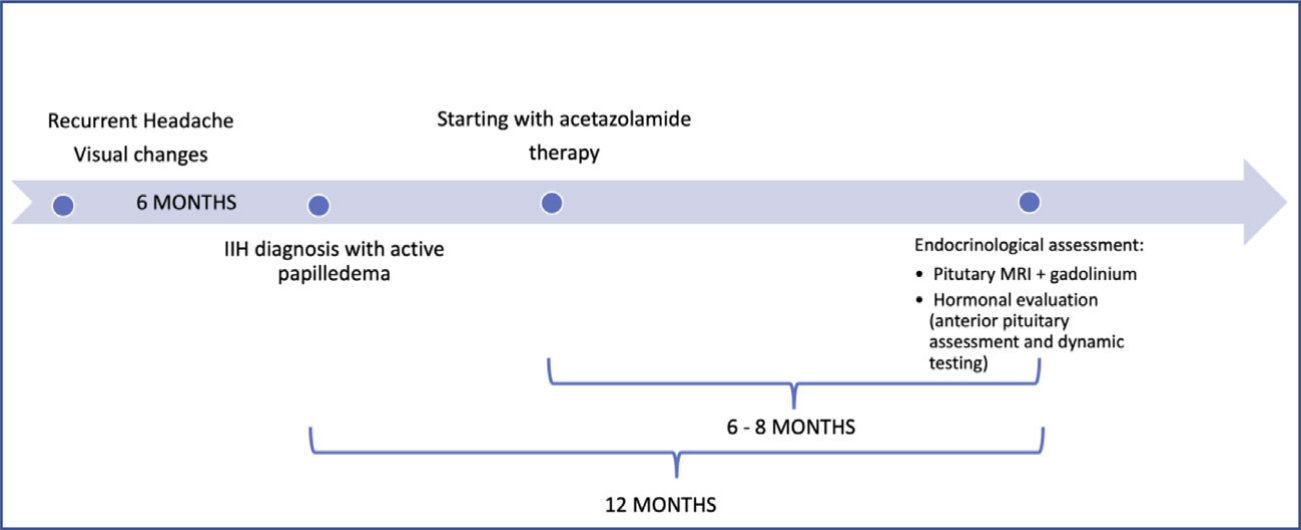
Timeline of the endocrinological evaluation in relation to IIH diagnosis and pharmacological treatment.

Prior to the enrolment, all patients underwent the routine diagnostic work-up for IIH diagnosis, which includes an MRI to exclude possible malignant causes of raised intracranial pressure, and lumbar puncture to measure the CSF opening pressure. No malignancies were detected, while two IIH typical alterations were reported (enlarged optic nerve sheath in the 79% of cases and descent of the cerebellar tonsils below the occipital hole in 50% of cases). All patients showed an increased CSF opening pressure (>25 cm H_2_O). At the time of our first evaluation, all patients had been diagnosed within the preceding 12 months with IIH according to the modified Dandy criteria: a) symptoms, if present, of increased intracranial pressure or papilledema; b) signs of increased intracranial pressure or papilledema; c) elevated intracranial pressure upon lumbar puncture with normal cerebrospinal fluid composition; d) no evidence of ventriculomegaly, mass, structural, or vascular lesion on magnetic resonance imaging or contrast-enhanced computed tomography for typical patients, and magnetic resonance imaging and magnetic resonance venography for all others; e) no other cause (including medication) of intracranial hypertension (15). Because of the presence of active papilledema, the patients had been started on pharmacological treatment with acetazolamide (500–750 mg/day) for the preceding 6–8 months (**Figure 1**). All patients tolerated diuretic treatment with no adverse event or weight loss reported, and a good control of headaches not requiring additional pain management. None of them presented cardiovascular disease (NYHA III-IV), hepatic and renal affections or pregnancy at the time of the enrolment.

The general characteristics of the fifteen patients are summarized in **Table 1**. Briefly, BMI ranged from 22.67 to 51.44 kg/m^2^ with a strong prevalence of overweight/obesity (87%): two patients had a normal weight, one patient was overweight, and 12 patients were frankly obese. Regarding other risk factors, the median number of previous pregnancies was 1.50 ± 1.40, 10 patients were of childbearing age, while five patients out of 15 were menopausal. Endocrinological findings consisted of one case of sublicnical hyperthyroidism due to suppressive hormone replacement therapy following total thyroidectomy for papillary thyroid carcinoma and two cases of mild hirsutism with disturbances of the menstrual cycle, suggesting polycystic ovary syndrome (PCOS). All other women of childbearing age reported regular menstrual cycles.

**Table 1:** General characteristics of the study population.

General characteristics (n = 15)	Mean ± SD or n (%)
Age (years)	42.13 ± 12.97
Menarcheal age (years)	11.80 ± 1.31
Pregnancies (number)	1.50 ± 1.40
BMI (kg/m²)	35.16 ± 8.21
Obesity/overweight (n/%)	13 (87)
Obesity duration (years)	11.71 ± 7.65

IIH, Idiopathic Intracranial Hypertension; BMI, Body mass index. Data are shown as mean ± SD or n (%).

## Diagnostic Assessment

All patients had medical history collected, physical examination and laboratory work performed (hematology, biochemistry, and dynamic tests when appropriate) as part of the routine initial evaluation that all patients accessing the Day Service of the High Specialization Center for the Care of Obesity (CASCO) undergo. Anthropometric parameters such as weight (kg), height (m), and BMI (kg/m^2^) were obtained between 8.00 and 10.00 a.m. in fasting subjects with an empty bladder, wearing light clothing and no shoes. Blood samples were collected at 8.00 a.m. and measurement of basal anterior pituitary function included: growth hormone (GH) (ng/ml) and IGF-1 (ng/ml) measured by enzyme-labeled chemiluminescent immunometric assay (Immulite XPi 2001/2000, Siemens); TSH (µUI/ml), free T3 (pg/ml), free T4 (ng/dl), FSH (mUI/ml), LH (mUI/ml), estradiol (pg/ml), dehydroepiandrosterone sulfate (DHEA-S) (µg/ml), and prolactin (PRL, ng/ml) by means of a chemiluminescent microparticle immunoassay (CMIA, Architect System; Abbott Laboratories, IL, USA). Serum ACTH (pg/ml) was assessed by immunoradiometric assay (IRMA, Cisbio Assays, France), serum cortisol (ng/ml) was measured by radio immune assay (RIA, Beckman Coulter); 24-h urinary free cortisol (ng/24 h) was also assayed (RIA, Beckman Coulter). The GH pituitary reserve was evaluated in patients with partial or complete ES according to the AACE guidelines and diagnostic criteria for GH deficiency (GHD). The procedure involved the administration of 1 μg/kg of GHRH (Ferring) intravenous bolus and 0.5g/kg of L-arginine hydrochloride 30% intravenous infusion in 30 min; blood was collected for GH at 0, 30, 45, 60, and 90 min, as previously described (16). GHD was diagnosed when peak levels were ≤ 8.0 or ≤ 4.0 µg/L for overweight or obese patients, respectively; GH -peak ≤11.0 µg/L was considered diagnostic for normal-weight subjects. Furthermore, we calculated the area under the concentration–time curve (AUC) by the trapezoidal method to assess the GH secretory capacity. Patients with low serum cortisol were further investigated with an ACTH stimulating test (Synachten, 0.25 mg/1 ml IV), blood samples for cortisol were collected 30 and 60 min following the injection. All blood samples were transferred to the local laboratory and handled according to the relative standards of practice.

**Figure 2:**
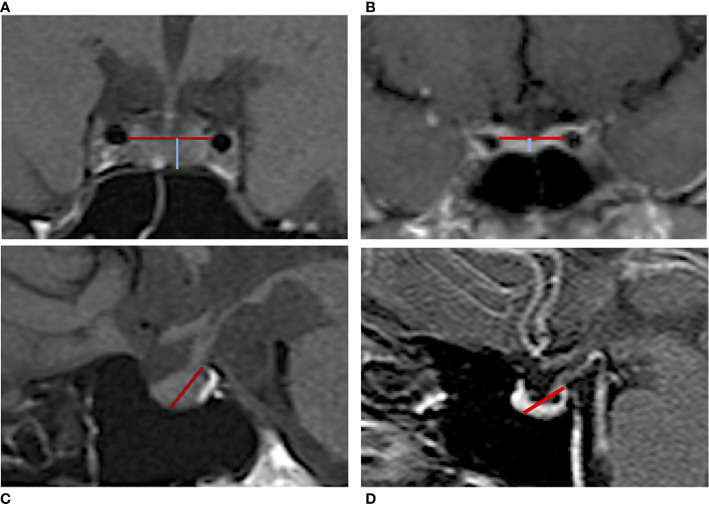
Manual measurement of the pituitary gland in MRI T1-weighted images. Coronal scan of an IIH patient **(A)** without ES and **(B)** with ES. The blue line reflects the gland height (cranio-caudal diameter) defined as the distance between the inferior edge of the *sella turcica* and the superior edge of the pituitary gland; the red line indicates the pituitary gland thickness (latero-lateral diameter) that was measured as the maximum transverse diameter. Sagittal scan of an IIH patient **(C)** without ES and **(D)** with ES. The red line indicates the pituitary length (anterior-posterior diameter) measured from the posterior clinoid process to the middle point of the pituitary fossa.

Pituitary morphology was assessed through MRI, using 1.5 T scanners (Siemens, Erlangen, Germany), in high-resolution, T1-weighted sequences. Length and area measurements of the pituitary gland and sellar compartment were obtained by manual delineation on the mid-coronal MR images and on mid-sagittal scans by two independent, highly experienced, observers. The observers were blinded to subject information. Height measurements were performed along the direction of the pituitary stalk for improved consistency. Pituitary gland height (cranio-caudal diameter, CC) was defined as the distance between the inferior edge of the *sella turcica* and the superior edge of the pituitary gland, measured immediately sideways to the stalk; pituitary gland thickness (latero-lateral diameter, LL) was measured as the maximum transverse diameter of the pituitary in coronal T1-WI showing the pituitary stalk (**Figures 2A, B**). In order to complete the assessment of the pituitary gland given the several physiological variations in its size and shape, we also evaluated the pituitary length in sagittal T1W scans, defined as the maximum antero-posterior extent of the gland in horizontal plane (anterior-posterior diameter, AP) (**Figures 2C, D**). The pituitary volume (PV, mm^3^) was calculated according to the formula V = (AP x LL x CC) mm* 0.523/2. Partial ES diagnosis was made when less than 50% of the sella was filled with cerebrospinal fluid (CSF) and the pituitary gland height was ≥3 and <5 mm; total ES when more than 50% of the sella was filled with CSF, and the pituitary gland height was ≤3 mm. All study participants underwent an eye examination performed by an ophthalmologist, and the presence of papilledema was recorded.

All patients were in good health and no neurological signs were detected upon examination. The biochemical assessments are reported in **Table 2**. TSH, free T4, and free T3 levels were within the normal ranges in twelve patients: one patient had iatrogenic subclinical hyperthyroidism due to suppressive levotiroxine therapy for high risk carcinoma follow-up, other two subjects were diagnosed with subclinical primary hypothyroidism due to Hashimoto’s thyroiditis. Regarding the gonadotropic axis, we evaluated it exclusively in premenopausal women (10/15 patients) during the follicular phase and no abnormality was detected. Three obese patients aged 16, 19, and 45 showed relatively high levels of LH over FSH (17.3, 13.3, 13.4, and 5 mUI/ml and 5.9 and 6.00 mUI/ml, respectively); moreover, the youngest patient reported menstrual irregularities. PRL concentration were normal apart from two patients with PRL levels ​​above the upper normal limit (34.4 and 29.3 ng/ml) in absence of elevated TSH, with no clinical significance, as no potentially related signs or symptoms were present. Two subjects showed low serum cortisol (61.6 ng/ml; 55 ng/ml), low ACTH (18.5; 17.5 respectively) with normal 24-h urinary cortisol and were therefore further investigated with an ACTH stimulating test. Both patients showed a normal response (peak value > 185 ng/ml), excluding possible adrenal insufficiency. One patient reported low cortisol (90 ng/ml) in relation to low ACTH values (8.7 pg/ml), but she was on steroid treatment for acute lumbosciatalgia. DHEA-S was increased in two patients suffering from mild hirsutism, obesity and oligomenorrhea in the preceding 2 months, one of which had also a high LH/FSH ratio, suggestive of possible PCOS. GH and IGF-1 levels were within the age-adjusted ranges, except for one 42-year-old obese patient showing plasma IGF-1 levels just below the lower reference limit (89 ng/ml, r.v. 96–288 ng/ml), who however reported a normale response to the dynamic test (peak GH concentration of 15.87 ng/ml at 45 min). No GHD cases were detected in the eleven patients affected by ES who underwent a GHRH+Arg test. More specifically, GH response showed a high variability, but all of the obtained peak values were frankly over the diagnostic cut-off: the minimum stimulated GH concentration in normal-weight, overweight and obese patients were 15.87, 8.62, and 6.49 ng/ml, respectively.

**Table 2:** Anterior pituitary basal and dynamic assessment in patients affected by Idiopathic Intracranial Hypertension.

Hormonal assessment	Mean ± SD	Reference values
TSH (µUI/ml)	3.47 ± 3.82	0.27 - 4.20
FT4 (ng/dl)	1.11 ± 0.11	0.70 -1.48
FT3 (pg/ml)	2.78 ± 0.42	1.71 - 3.71
FSH^a^	6.51 ± 4.49	3.5–12.5
LH^a^	9.20 ± 5.60	2.4–12.6
Estradiol (pg/ml)^a^	93.97 ± 91.01	35–169
DHEA-S (µg/ml)	1.84 ± 1.48	0.35–2.56
Serum cortisol (ng/ml)	134.4 ± 48.56	96.4–261
24 h-urinary cortisol (ng/24 h)	55.00 ± 19.12	13.77–75.4
ACTH (pg/ml)	21.82 ± 17.85	10–90
PRL (ng/ml)	17.23 ± 7.92	4.80–23.30
GH (ng/ml)	2.05 ± 3.50	0.05–16.00
IGF1 (ng/ml)	214.80 ± 97.47	_
IGF-1 SDS	-0.43 ± 2.26	_
GHRH + arginine test – AUC (ng/ml/min) ^b^	1280.68 ± 1550.26	_

a FSH, LH, Estradiol have been measured in 10 out of 15 patients of the study population (pre-menopausal women).

b GHRH+ Arg. test was administered only to patients with total or partial ES (11/15 IIH patients).

IIH, Idiopathic intracranial hypertension; TSH, Thyroid stimulating hormone; FT3, free Triiodothyronine; FT4, free Thyroxine; FSH, Follicular stimulating hormone; LH, Luteinizing hormone; DHEA-S, Dehydroepiandrosterone sulfate; ACTH, Adrenocorticotropic hormone; PRL, Prolactin; GH, Growth hormone; IGF1, Insulin-Like Growth Factor 1; SDS, Standard deviation score; AUC, Area under the curve. Data are shown as mean ± SD.

At MRI evaluation, ES was detected in 73% of IIH patients. In detail, five complete and six partial ES were recorded. Patients with normal pituitary diameters were younger than those with ES. Among the measured parameters, the coronal pituitary height showed values ​​close to the lower limit (mean ± SD, 4.53 ± 1.33 mm), while the mean coronal pituitary width was 15.08 ± 2.13 mm and the sagittal pituitary length was 11.49 ± 1.49 mm. Calculated PV showed a mean value of 389.20 mm^3^, ranging from 213.85 to 642.27mm^3^.

## Discussion

We herein confirm that female patients with overweight/obesity and IIH show a conserved pituitary function despite a 73% prevalence of ES. Whether a strong connection between ES and IIH has been well established, with previous literature reporting ES in 76–85% of subjects with IIH (3), the absence of hormonal alterations came unexpected, as ES itself may be a risk factor for pituitary secretory dysfunction.

Several studies conducted in patients suffering from primary ES showed a variable prevalence of hormonal dysfunctions. Mild hyperprolactinemia has been frequently reported (7–10% of the cases), mostly because of pituitary stalk compression or stretching as a consequence of the remodeling of the hypothalamic-pituitary region and altered CSF dynamics. The most frequent hormonal alteration appears to be GHD (4–57%) (17, 18), followed by gonadotropin deficiency (about 6%). A variable prevalence has been identified for secondary hypoadrenalism, hypothyroidism and hypogonadism, ranging from 2.3 to 32% of cases (17), whether panhypopituitarism is rare (about 2%) (9). Insufficiencies in multiple pituitary axes are more common, with an estimated prevalence of 30%, as opposed to isolated events, occurring in 21% of cases (19).

If ES is often associated with functional impairment, pituitary dysfunction has been reported rarely in IIH, with most available literature regarding pediatric populations. On the contrary, adults have been marginally screened. The only studies aimed at evaluating the functional integrity of the pituitary gland were conducted in the past by Reid et al. and Sorensen et al., reporting no significant alterations (12, 13). Consistent with these findings, we did not observe any major abnormalities of the anterior pituitary function in IIH patients. Even when comparing the GH peak and GH-AUC with other studies conducted in obese patient with ES (18), we noticed that the GH secretory capacity was preserved in our study population. We report slightly higher prolactin levels, not associated to a specific symptomatology, likely linked to pituitary stalk stretching. The gonadotropic axis was preserved where investigated, except for three patients who had a slightly altered LH/FSH ratio, together with increased DHEA-S values, suggesting a condition of mild hyperandrogenism. Noteworthy, PRL might have a role on adrenal steroidogenesis, possibly explaining the finding of higher PRL and DHEAS in these patients (20). Interestingly, Klein et al. (21) documented increased levels of circulating androgens including total and bioavailable testosterone and androstenedione in a cohort of 51 female patients with IIH, raising the possibility of a mechanistic link between androgen excess and IIH. Moreover, the authors observed a link between biochemical hyperandrogenism and earlier IIH onset, and previous studies reported an association between PCOS—the most common well-defined form of overt hyperandrogenism—and pseudotumor cerebri, supporting the hypothesis that the onset of IIH may be accelerated by androgen excess. However, the proposed association between PCOS and IIH could be due to that both conditions are more common in women with obesity of childbearing age, and mechanistic studies are required to further investigate the topic.

Noteworthy, Prabhat et al. recognized hormonal abnormalities in 37.5% of 80 patients with IIH. Briefly, they detected hypocortisolemia in 20%, hyperprolactinemia in 13.8%, hypothyroidism in 3.8%, hypogonadism in 1.25%, elevated levels of gonadotropins were found in 5% of cases. Moreover, they observed a negative correlation between hormonal alterations and partial empty sella (22). However, the patients were not under pharmacological treatment, and no dynamic testing was performed to confirm hormonal deficiency, possibly accounting for the different findings. Upon evaluation of the hormonal profile of our population based on the presence of ES, no substantial differences were observed nor in terms of clinical or biological parameters, nor in terms of treatment. Noteworthy, ES was not present in only four patients and they trended younger than the affected ones, although no reliable statistics could be performed given the very small sample size.

The absence of hormonal disorders could be related to the radiological evidence of an isolated reduction in coronal height of the gland, in the absence of any other measure alteration in the coronal and sagittal MRI scans. In pseudotumor cerebri, intracranial hypertension causes pituitary crushing and seems to lead to an enlarged distribution of the gland over the sella turcica; however, the pituitary parenchyma remains functional. In this regard, some authors have proposed to reserve the term “empty sella” only when pituitary is no longer visible. Our data are consistent with previous reports supporting the hypothesis that, despite the frequently observed ES, there may be no substantial loss of functional parenchyma, and the pituitary gland may be compressed rather than atrophic (3, 23). This hypothesis could justify why patients with IIH and ES do not show pituitary hormonal dysfunctions, unlike patients with ES uncoupled with IIH.

Noteworthy, the patients were being treated with acetazolamide, that has been shown to induce complete recovery of papilledema and improvement in the height of the midsagittal pituitary height, possibly affecting the observed results (11, 24). On the other hand, the re-expansion of the pituitary gland does not occur as rapidly as the resolution of papilledema, and in fact some authors propose to integrate the follow-up not only with the fundus oculi evaluation, but also with MRI, as the persistence of an ES and posterior globe flattening may be useful for IIH detection even after the resolution of papilledema and the normalization of ICP (25). It should also be emphasized that acetazolamide has not always proved effective in restoring the pituitary morphology, which was then achieved through a lumboperitoneal shunt (11) or serial lumbar punctures (26). Unfortunately, none of these studies evaluated pituitary function contextually. In support of the hypothesis that concomitant diuretic treatment might have affected our results, Prabhat et al. have reported higher rates of pituitary dysfunction in treatment naïve patients (22). As mentioned, some authors suggested that lumbar punctures may have an impact on the occurrence of headaches and may have a therapeutic role, similar to diuretics. Nevertheless, the few interventions performed in this sense did not produce significant advantages in terms of reducing intracranial pressure on long term or in relation to headaches relief (27, 28). Based on this, we believe the potential effect on pituitary morphology and function to be reasonably negligible, especially if the lumbar puncture is used only for diagnostic purposes. Conversely, obesity is associated with both high prevalence of ES and reduced pituitary volume, suggesting that chronic compression may ultimately lead to gland atrophy and subsequent reduced volume (5, 18, 29).

This study has some limitations. First, only a limited number of IIH patients were enrolled. Second, gonadotropins were only evaluated in women of childbearing age. Third, the evolution of the papilledema over time was unavailable, as well as follow-up data after acetazolamide discontinuation, and, in general, no longitudinal assessment was performed. However, given the virtual absence of literature regarding the topics explored in the adult population, our data may to be of interest for further studies. Therefore, we suggest, in line with other authors (5, 30), to evaluate pituitary function over time in order to exclude the development of hormonal deficiencies whose onset may be delayed and progress over time. This is likely even more crucial in subjects with obesity, as weight excess represents an additional risk factor for pituitary dysfunction, and, with the prevalence of obesity skyrocketing (31), the occurrence of patients with obesity and pituitary morphology/function derangements will likely increase accordingly.

In conclusion, we confirm a strong association between weight excess, ES and IIH. Our case series provides additional evidence that the radiological finding of ES in IIH patients could represent a compressed pituitary gland with a different parenchyma distribution within the sella turcica rather than real atrophy. This, along with the ongoing drug treatment, could explain the observed integrity of the hormonal axes, including the somatotropic one that is most frequently affected in ES patients. A longitudinal evaluation of the pituitary function in IIH patients and controlled studies are needed in order to understand aspects that are still unclear, e.g., the impact of acetazolamide discontinuation on pituitary volume and function.

## Data Availability Statement

The datasets generated for this study are available on request to the corresponding author. Requests to access the datasets should be directed to carla.lubrano@uniroma1.it.

## Ethics Statement

Ethical review and approval was not required for the study on human participants in accordance with the local legislation and institutional requirements. Written informed consent to participate in this study was provided by the participants’ legal guardian/next of kin. Written informed consent was obtained from the individual(s), and minor(s)’ legal guardian/next of kin, for the publication of any potentially identifiable images or data included in this article.

## Author Contributions

RT, AC, EG, and AB acquired the data. CL, EP and SM analyzed and interpreted data. RT wrote the manuscript. CL, MW, AM, and LG revised it. All authors contributed to the article and approved the submitted version.

## Conflict of Interest

The authors declare that the research was conducted in the absence of any commercial or financial relationships that could be construed as a potential conflict of interest.

## Abbreviations

ACTH, Adrenocorticotropic hormone; AUC, Area under the curve; BMI, body mass index; CSF, cerebrospinal fluid; DHEA-S, Dehydroepiandrosterone sulfate; ES, empty sella; FSH, Follicular stimulating hormone; FT3, free triiodothyronine; FT4, free thyroxine; GH, Growth hormone; IGF1, Insulin-Like Growth Factor 1; IIH, Idiopathic intracranial hypertension; LH, Luteinizing hormone; MRI, magnetic resonance imaging, PCOS, Polycystic ovary syndrome; PRL, Prolactin; PV, pituitary volume; SDS, Standard deviation score; TSH, Thyroid stimulating hormone.
